# Novel Routes for Improving Biocontrol Activity of *Bacillus* Based Bioinoculants

**DOI:** 10.3389/fmicb.2015.01395

**Published:** 2015-12-10

**Authors:** Liming Wu, Hui-Jun Wu, Junqing Qiao, Xuewen Gao, Rainer Borriss

**Affiliations:** ^1^College of Plant Protection, Nanjing Agricultural University, Key Laboratory of Monitoring and Management of Crop Disease and Pest Insects, Ministry of AgricultureNanjing, China; ^2^Institute of Plant Protection, Jiangsu Academy of Agricultural SciencesNanjing, China; ^3^Fachgebiet Phytomedizin, Institut für Agrar- und Gartenbauwissenschaften, Humboldt-Universität zu BerlinBerlin, Germany; ^4^Nord Reet UGGreifswald, Germany

**Keywords:** plant growth-promotion, biocontrol, *Bacillus amyloliquefaciens* subsp. *plantarum*, mersacidin, bacillomycin D, surfactin, bacilysin, harpin genes

## Abstract

Biocontrol (BC) formulations prepared from plant-growth-promoting bacteria are increasingly applied in sustainable agriculture. Especially inoculants prepared from endospore-forming *Bacillus* strains have been proven as efficient and environmental-friendly alternative to chemical pesticides due to their long shelf life, which is comparable with that of agrochemicals. However, these formulations of the first generation are sometimes hampered in their action and do not fulfill in each case the expectations of the appliers. In this review we use the well-known plant-associated *Bacillus amyloliquefaciens* type strain FZB42 as example for the successful application of different techniques offered today by comparative, evolutionary and functional genomics, site-directed mutagenesis and strain construction including marker removal, for paving the way for preparing a novel generation of BC agents.

## Introduction

As stated by Compant et al. (2005) in their excellent review, pathogenic microorganisms affecting plant health are a major and chronic threat to food production and ecosystem stability worldwide. Approximately 25% of the world’s crop yield is lost every year due to plant pathogens (Lugtenberg, 2015). As agricultural production intensified over the past few decades, producers became more and more dependent on agrochemicals as a relatively reliable method of crop protection helping with economic stability of their operations (Schäfer and Adams, 2015).

However, due to the negative impact on environment caused by agrochemicals, disease control by beneficial bacteria as an alternative to chemical pesticides in plant protection is steadily increasing and begins to replace in part chemical pesticides (Qiao et al., 2014). It has been shown that applying spore formulations of the plant-beneficial bacterium *Bacillus amyloliquefaciens* does not affect the composition of rhizosphere microbial community (Chowdhury et al., 2015a). An increasing number of farmers are recognizing the need for other avenues for pest control that are not as damaging to the environment and the land. According to a comprehensive study of BCC Research, global markets for biopesticides will grow from USD54.8 billion in 2013 to USD83.7 billion to 2019^1^.

Thereby, biological preparations from spore-forming *Bacillus* sp. are preferred, because their long-term viability facilitates the development of commercial products (Borriss, 2015a). These plant-associated bacteria are characterized by their simultaneous plant-growth promoting (PGP) and biocontrol (BC) activity. It should be mentioned here, that both features are linked with each other and should not artificially separated by regulatory authorities: Plant growth promoting effects strengthen plants and made them more resistant against pathogens and *vice versa* suppression of pathogens enhances plant health and reduces harvest losses (Kamilova et al., 2015). Unfortunately, the success of such biologicals in agriculture is still hampered by sometimes inconsistent field performance due to insufficient knowledge about basic mechanisms of interactions between bacilli and plants, although some progress has been made in last decade (Ravensberg, 2015).

Plant-associated *B. amyloliquefaciens* strains belonging to subsp. *plantarum* (*methylotrophicus*) (Borriss et al., 2011; Dunlap et al., 2015) are distinguished from other representatives of endospore-forming *B. amyloliquefaciens* by their ability to colonize plant rhizosphere, to stimulate plant growth and to suppress competing phytopathogenic bacteria and fungi. Due to their biofertilizer and BC properties they are becoming increasingly important as a natural alternative to chemical pesticides and other agrochemicals (Borriss, 2011). We have directed our research on *B. amyloliquefaciens* FZB42^T^, the type strain for *B. amyloliquefaciens* subsp. *plantarum*. Since its first description (Krebs et al., 1998) more than 70 articles dealing with FZB42 have been published^2^. In order to reveal the specific genomic features linked with the properties beneficial for plant growth and BC, we have sequenced the whole genome of FZB42 as the first example of Gram-positive plant beneficial bacteria (Chen et al., 2007).

Comparative genome analysis, transposon mutagenesis, transcriptome and proteome analysis of this model organism have been proven as valuable means to analyze its plant growth promoting and BC activities (Chowdhury et al., 2015a). Ten giant gene clusters covering nearly 10% of the whole genome and responsible for non-ribosomal and ribosomal synthesis of secondary metabolites with antimicrobial and nematocidal action were identified (Borriss, 2013). In addition, an incomplete gene cluster directing immunity against the type B lantibiotic mersacidin was detected (**Table 1**). In this review we will describe several possibilities offered today by *in vitro* techniques for enhancing the beneficial action of bioformulations based on *B. amyloliquefaciens* FZB42, and its close relatives SQR9 and NJN6, isolated by the laboratory of Qirong Shen, Nanjing Agriculture University.

**Table 1:** Genes and gene cluster encoding for secondary metabolites in *Bacillus amyloliquefaciens plantarum* FZB42.

Metabolite	Genes and gene cluster	Size (bp)	Genome position (bp)	MIBiG	Effect against	Reference
**Sfp-dependent non-ribosomal synthesis of lipopeptides (NRP)**					
Surfactin	*srfABCD*	28,544	341,664-370,208	BGC0000433	virus, *Mycoplasma*	Koumoutsi et al., 2004
Bacillomycin D	*bmyCBAD*	39,113	c1,908,427-c1,869,309	BGC0001090	fungi	Koumoutsi et al., 2004
Fengycin	*fenABCDE*	37,669	c1,968,997-c1,931,328	BGC0001095	fungi	Koumoutsi et al., 2004
Bacillibactin	*dhbABCDEF*	11,954	c3,032,970-c3,021,016	BGC0001185	microbial competitors	Chen et al., 2007
**Sfp-dependent non-ribosomal synthesis of polyketides (PKS)**					
Macrolactin	*mlnABCDEFGHI*	53,253	1,391,841-1,445,094	BGC0000181	bacteria	Schneider et al., 2007
Bacillaene	*baeBCDE, acpK, baeGHIJLMNRS*	72,437	1,700,345-c1,772,782	BGC0001089	bacteria	Chen et al., 2006
Difficidin	*dfnAYXBCDEFGHIJKLM*	69,523	c2,276,743-c2,346,266	BGC0000176	bacteria	Chen et al., 2006
**Sfp-independent non-ribosomal synthesis (NRP)**					
Bacilysin	*bacABCDE, ywfG*	5,907	c3,593,877-c3,599,784	BGC0001184	bacteria	Chen et al., 2009b
**Ribosomal synthesis of processed and modified peptides (bacteriocins, lantibiotics, RiPPs)**					
Plantazolicin	*pznFKGHIAJC DBEL*	9,891	726,457-736,348	BGC0000569	*B. anthrax*, nematodes	Scholz et al., 2011
Amylocyclicin	*acnBACDEF*	4,112	c3,048,678-c3,044,568	BGC0000616	related bacteria	Scholz et al., 2014
Mersacidin (partial)	*mrsK2R2FGE*	4,828	c3,774,552-c3,769,734	BGC0000527	Gram-+ bacteria	Borriss, 2013


*The available MIBiG entries (Medema et al., 2015) are indicated.*

## Phylogenomics of *Bacillus amyloliquefaciens*

**FIGURE 1:**
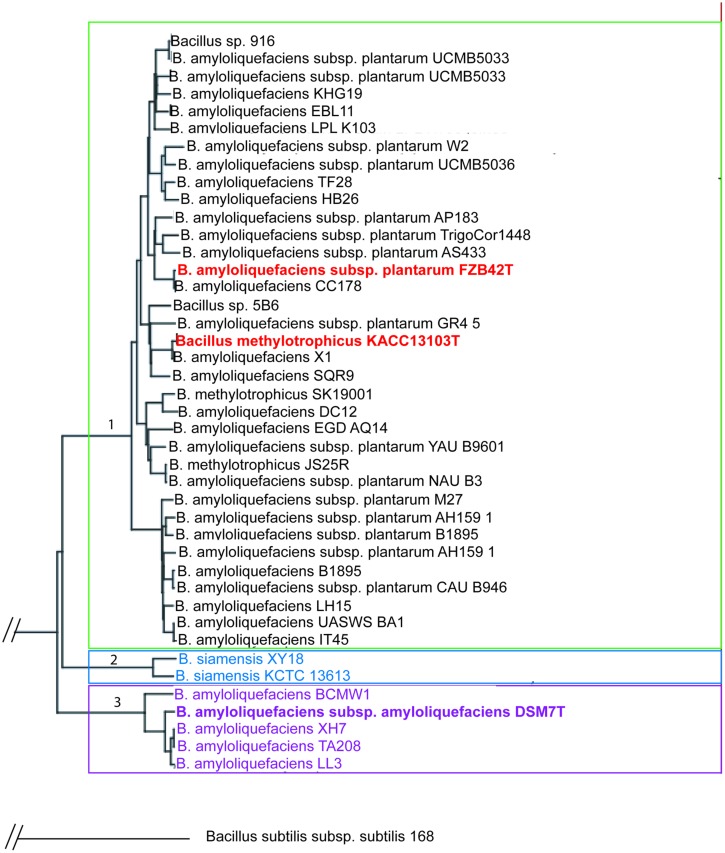
**Phylogenetic tree of *Bacillus amyloliquefaciens* chromosomes currently available in public databases.** Based on the core genome of 2104 CDS the divergence of the plant-associated bacteria (*B. amyloliquefaciens* subsp. *plantarum*) and *B. siamensis* and *B. amyloliquefaciens* subsp. *amyloliquefaciens* was quantified with FZB42T employed as reference to construct the tree according to Blom et al. (2009). Every set of orthologous genes found in all genomes was separately aligned using the multiple alignment tool MUSCLE (Edgar, 2004). The alignments were concatenated to one huge multiple alignment. A distance matrix was calculated from this alignment and finally a phylogenetic tree was constructed based on this distance matrix using the Neighbor-Joining method. The two latter methods are used in the PHYLIP implementations by Felsenstein (http://evolution.genetics.washington.edu/phylip.html). The Neighbor-Joining method was chosen as it is a heuristic approach with a very good computational efficiency, making it well suited for large datasets resulting from the core genome based tree construction.

The genus *B. amyloliquefaciens* harbors members of different ecotypes (plant-associated and non-plant associated, Reva et al., 2004). Our analysis based on the use of all core genes of a set of 42 genomes to maximize the sequence support for the phylogenetic tree (Zdobnov and Bork, 2007) and used the pipeline provided by the EDGAR software (Blom et al., 2009). According to phylogenomic analysis *B. amyloliquefaciens* is clustered into three taxonomic units which could be considered as ‘subspecies’ (**Figure 1**):

(1)  *B. amyloliquefaciens* subsp. *plantarum* (*B. methylotrophicus*)
(2)  *B. amyloliquefaciens* subsp. *siamensis* (*B. siamensis*)
(3)  *B. amyloliquefaciens* subsp. *amyloliquefaciens*

Interestingly, the two available genomes of *B. siamensis* formed a separate taxonomic unit within the *B. amyloliquefaciens* subspecies complex suggesting that the taxonomic classification of *B. siamensis* has to be reconsidered. As reported recently (Dunlap et al., 2015), *B. methylotrophicus* and *B. amyloliquefaciens* subsp. *plantarum* are not distinguishable by their core genome sequences and form together a robust taxonomic unit comprising all plant-associated representatives of the genus *B. amyloliquefaciens* (group 1). Overall, the *B. amyloliquefaciens* pan genome consists of 8652 CDS, whilst the core genome consists of 2104 CDS with *Bacillus amyloliquefaciens* FZB42 (NC_009725) used as reference.

The pan genome derived only from representatives of *B. amyloliquefaciens* subsp. *plantarum* and *B. methylotrophicus* (plant-associated group 1) comprises 7936 CDS, which is reflecting the high flexibility in adapting to plant-associated lifestyle. The core genome formed by the 35 *B. amyloliquefaciens* subsp. *plantarum* and 3 *B. methylotrophicus* genomes consists of 2295 CDS suggesting that 54 genes of the core genome are unique for the subsp. *plantarum* (*B. methylotrophicus*) and do not occur in the non-plant-associated subsp. *amyloliquefaciens* and in *B. siamensis* (Qiao et al., 2014). Within these singletons are the genes involved in non-ribosomal synthesis of the polyketides difficidin (Chen et al., 2006) and macrolactin (Schneider et al., 2007), an iturin-like compound (e.g., bacillomycin or iturinA, Borriss et al., 2011), and several genes involved in carbohydrate degradation and transport, such as glucuronate isomerase (*uxaC*), 2-keto-3-deoxygluconokinase (*kdgK*), 2-keto-3-deoxygluconate -6-isomerase-6-phosphate aldolase (*kdgA*), endo-1,4-beta-glucanase (*eglA*), and saccharifying amylase (*amyE*). Many of these genes, unique for plant-associated *B. amyloliquefaciens* seem to be acquired by horizontal gene transfer. FZB42 contains 17 genomic islands (Chen et al., 2007). Certain DNA islands appear to be linked with the plant-associated lifestyle. Island 7 (28,754 bp) for instance, contains genes with striking similarity to genes involved in extracellular arabinogalactane hydrolysis, galactose uptake by a sugar-specific phosphotransferase system IIABC and galactose catabolism in enterococci, lactobacilli and *Erwinia carotovora* (Chen et al., 2007). It can be assumed that acquisition of this molecular toolbox, comprising several elements derived from other soil- and plant-associated bacteria has enhanced the ability of FZB42 to exploit plant-derived polysaccharides.

A recent comparative analysis of core genomes from 28 *B. amyloliquefaciens* subsp. *plantarum* and 32 *B. amyloliquefaciens* species identified 193,952 bp of sequences that are present within the subsp. *plantarum* core genome but absent in the *B. amyloliquefaciens* core genome (Hossain et al., 2015). Among these genetic loci there were 73 genes shared by all 28 *plantarum* strains but were not present in any strains of subsp. *amyloliquefaciens.* The putative functions of these genes included transportation (7 genes), regulation (7 genes), signaling (1 gene), carbon degradation (10 genes), synthesis of secondary metabolites (19 genes), and hypothetical proteins (12 genes). Hossain et al. (2015) hypothesized that some of these gene products may be involved in interactions with plants.

Genes involved in ribosomal synthesis of several bacteriocins, such as mersacidin (Borriss, 2013), plantazolicin (Scholz et al., 2011), and amylocyclicin (Scholz et al., 2014), were detected in several representatives of *B. amyloliquefaciens* subsp. *plantarum*, but are not part of the *plantarum* core genome. We hypothesize that most of the genes, unique in subsp. *plantarum* are involved in plant-bacteria interactions and in suppressing plant pathogens.

## *Bacillus amyloliquefaciens* subsp. *plantarum (methylotrophicus)* Fzb42^T^

**FIGURE 2:**
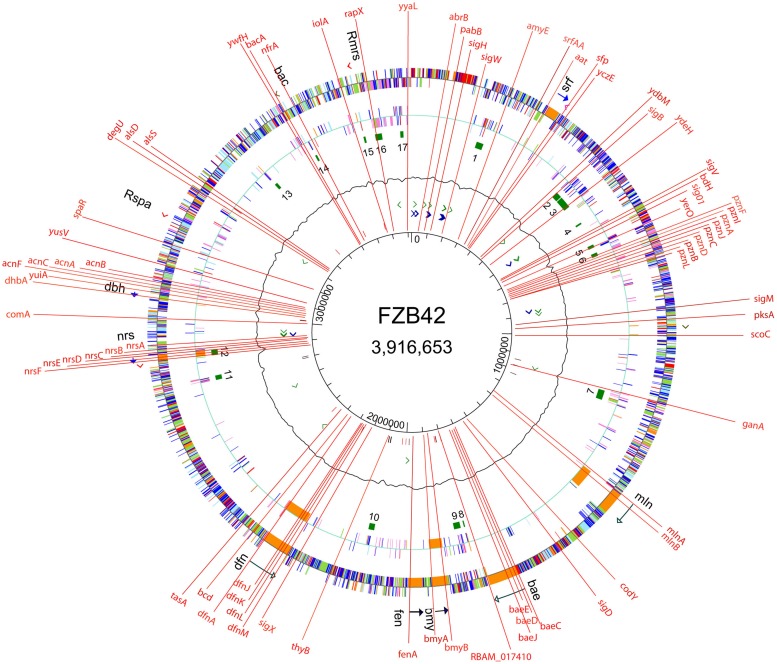
**Site directed mutations introduced into the genome of FZB42.** Mutants impaired in synthesis of secondary metabolites (non-ribosamal sythesis, *sfp*, surfactin, *srf*, plantazolizin, *pzn*, macrolactin, *mln*, bacillaen, *bae*, bacillomycin D, *bmy*, fengycin, *fen*, difficidin, *dfn*, unidentified peptide, *nrs*, siderophore, *dhb*, amylocyclicin, *acn*, bacilysin), volatiles (*alsS*, *alsD*, *bdh*), sugars (*ganA, iolA*), amylase (*amy*E), global regulators (*abrB*, *scoC*, *degU, codY*), alternative sigma factors (*sigH, sigW, sigB, sigV, sig01, sigM, sigD, sigX*), competence (*comA*), biofilm formation (*srfA, tasA*), oxidative stress response (*nfrA*), plant growth promotion (*RBAM-017410*), auxotrophy (*pabB*), and others (*yczE, ydbM, ydeH, yerO, yuiA, yusV, spaR, ywfH, rapX, yyaL*).
(4)  FZB42 is closely related to other BC *Bacilli* with industrial importance (FZB24, QST713, GB03, D747, MB1600, GA1, SQR9, NAUB3, YAU B9601), which are often wrongly assigned as being *B. subtilis*, but are also belonging to the same subspecies *plantarum* (*methylotrophicus*) as FZB42 (Borriss et al., 2011). Studies performed with FZB42 and its derivatives are therefore of general interest and valuable for understanding the mechanisms of action in this important group of endospore-forming plant-associated bacteria.

We have proposed to choose FZB42^T^ as model strain for plant-associated PGP and BC Bacilli for the following reasons (Borriss, 2011):

(1)  The strain is available for scientific research from public strain collections (BGSC 10A6 and DSM23117), despite that the strain is commercialized by ABiTEP GmbH Berlin and successfully applied in agri- and horticulture^3^.
(2)  The whole genome sequence of FZB42^T^ has been determined in 2007, as the first representative of gram-positive BC bacteria. Its 3,918-kb genome, lacks extended phage insertions, which occur ubiquitously in the related *Bacillus subtilis* 168 genome (Chen et al., 2007). Nearly 10% of the genome is devoted to synthesizing antibiotics, siderophores and bacteriocins (Chen et al., 2009a; Borriss, 2013).
(3)  In contrast to most environmental *Bacillus* strains, FZB42 is naturally competent and amenable to genetic transformation using a modified one-step protocol (Idris et al., 2007). In order to assign unknown gene functions, we generated more than 200 mutant strains targeted in 74 different genes involved in synthesis of secondary metabolites, volatiles, biofilm formation, alternative sigma factors and global transcription regulators (**Figure 2**). Moreover, strain derivatives of FZB42 were labeled by stable chromosomal integration of the green fluorescent protein (GFP+). Those strains were found extremely useful for studying root colonization after bacterial inoculation (Fan et al., 2011; Chowdhury et al., 2015b). The engineered mutant strains can be ordered from the Nord Reet UG Greifswald, Germany^4^.

## PGPR Bacilli Engineered for Enhanced Efficiency

A first step in improving efficiency of BC bacilli is identification of target genes involved in BC and root colonization. As stated above, the FZB42 genome harbors a rich arsenal of genes probably involved in synthesis of antimicrobial compounds. By applying a combined approach using gene knock-out mutants and chemical mass spectroscopy as analytic tools, we identified during last decade a total of 10 gene clusters involved in Sfp-dependent non-ribosomal synthesis of defined cyclic lipopeptides, c-LPs (4) and polyketides (3), Sfp-independent non-ribosomal synthesis of bacilysin, and ribosomal synthesis of the highly modified bacteriocins plantazolicin and amylocyclicin (Chowdhury et al., 2015a).

### Identification of Target Genes to Improve the Efficiency of PGPR Bacilli

#### Biocontrol

Several case studies using site-directed mutants were performed in order to demonstrate the antibacterial effect exerted by the polyketide difficidin and the dipeptide bacilysin. Difficidin was characterized as a highly unsaturated 22-membered macrocyclic polyene lactone phosphate ester (Wilson et al., 1987), and bacilysin, consisting of non-proteinogenic L-anticapsin and N-terminal L-alanine, was first isolated from *B. subtilis* (Kenig and Abraham, 1976). FZB42 was found efficient against the gram-negative pathogen *E. amylovora*, the causative agent of fire blight, a serious disease of orchard trees. Surprisingly, a mutant strain blocked in the production of difficidin (CH8 *Δdfn*) suppressed fire blight disease nearly in the same range as wild type FZB42. Moreover, a *sfp* mutant strain (CH3 *Δsfp*) unable to synthesize non-ribosomally lipopeptides and polyketides did still suppress growth of *E. amylovora*, suggesting that besides action of polyketides another antagonistic principle exist. In contrast, a double mutant impaired in non-ribosomal synthesis and bacilysin (RS06 *Δsfp Δbac*) was unable to suppress *E. amylovora* indicating that the additional inhibitory effect is due to production of bacilysin (Chen et al., 2009b).

**FIGURE 3:**
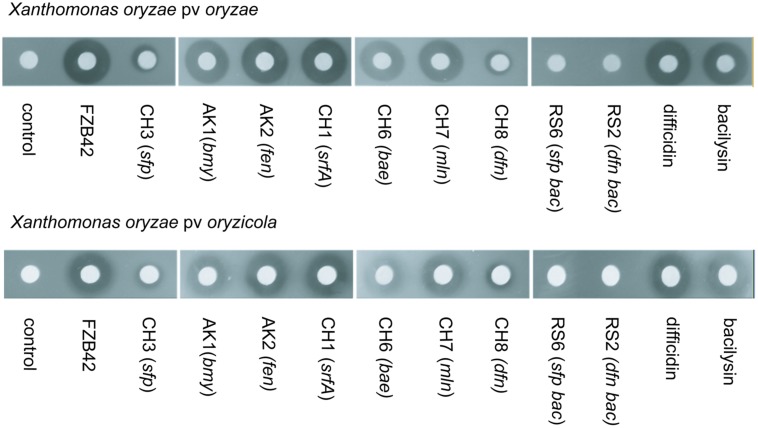
**Suppression of rice pathogens by FZB42 and its mutant strains.** **(Top)** agar diffusion assay using *Xanthomonas oryzae* pv.*oryzae* as indicator strain. **(Bottom)** agar diffusion assay using *Xanthomonas oryzae* pv. *oryzicola* as indicator strain. Bacilysin and difficidin, FZB42 and several mutant strains not impaired in bacilysin and difficidin synthesis have a clear antaogonistic effect against both pathogens whilst mutant strains RS02 and RS06, deficient in synthesis of bacilysin and difficidin, were unable to suppress both pathovars of *X. oryzae*. Mutant strains unable to synthesize difficidin but able to synthesize bacilysin (CH3, CH8) were found clearly repressed in their antagonistic action.

A similar study using appropriate mutant strains of FZB42 was performed recently, demonstrating that difficidin and bacilysin are also efficient against two different *Xanthomonas oryzae* pathovars, causative agents of damaging rice diseases (bacterial blight and bacterial leaf streak). Agar diffusion tests performed with several FZB42 mutant strains (**Figure 3**) revealed that the inhibitory effect of mutant CH8 *(Δdfn*) deficient in production of difficidin was clearly reduced compared to wild type FZB42. The double mutant RS06 (*Δsfp Δbac*) was completely unable to suppress *X. oryzae* pv *oryzae* and *X. oryzae* pv *oryzicola* suggesting that difficidin and bacilysin act as antagonists of the pathogenic *Xanthomonas* strains (Wu et al., 2015a).

Among 24 strains, *B. amyloliquefaciens* FZB42 showed the strongest bactericidal activity against the cyanobacterium *Microcystis aeruginosa*, the causative agent of harmful algal blooms in freshwater lakes and rivers. Surprisingly, the site-directed *sfp* mutant CH03, impaired in Sfp-dependent non-ribosomal synthesis of lipopeptides and polyketides including difficidin, was able to inhibit growth of *M. aeruginosa* in the same magnitude as the wild type. Random transposon mutagenesis using the mariner transposon TnYLB-1 revealed that mutant strains bearing transposon insertions within the *aroA* and *aroE* gene failed completely to inhibit *M. aeruginosa*. Products of the *aro* genes are involved in synthesis of aromatic amino acids and it is known that all *aro* mutants are impaired in bacilysin synthesis. Therefore, a knock-out mutation within the *bacB* gene was constructed and as expected the mutant was unable to inhibit growth of *M. aeruginosa* suggesting that bacilysin is responsible for inhibition (Wu et al., 2014).

#### Induced Systemic Resistance (ISR)

Plant defense triggered by surfactin, microbial volatile organic compounds (mVOCs) and other hitherto unidentified compounds is a main factor in suppressing plant pathogens by plant-associated bacteria (Pieterse et al., 2014). Selected plant-associated Bacillus *strains* emit mVOCs consisting of 2,3 butandiol and acetoin that can elicit plant defense (Ryu et al., 2004). Synthesis of 2,3 butandiol from pyruvate via 2-acetolactate and acetoin is governed by the products of the *alsS*, *alsD*, and *bdhA* genes in *B. subtilis* (Renna et al., 1993). *B. amyloliquefaciens* NJN-6 produces volatile compounds (VOCs) that inhibit the growth and spore germination of *Fusarium oxysporum* f. sp. *cubense*. Among the total of 36 volatile compounds detected, 11 compounds completely inhibited fungal growth. The antifungal activity of these compounds suggested that VOCs can play important roles over short and long distances in the suppression of *Fusarium oxysporum* (Yuan et al., 2012). However, except acetoin and 2,3 butandiol, the genes responsible for synthesis of the volatiles are unknown.

#### Root Colonization

A necessary precondition for the PGP and BC action of plant beneficial bacteria is their root colonization activity (Lugtenberg et al., 2001). In contrast to *Pseudomonas fluorescens* and some other gram-negative bacteria, bacilli are known as comparable “weak” colonizers of plant roots, and PGP bacilli are hardly to detect later than 3 months after their application (Bais et al., 2004; Chowdhury et al., 2013).

After identifying genes involved in root colonization and other plant-bacteria interactions, gene targeting techniques are useful techniques in order to generate strains with enhanced rhizosphere competence, given that no additional resistance genes are introduced. Enhanced root colonization and BC activity was gained in *B. amyloliquefaciens* SQR9 by disruption of the *abrB* gene encoding a global regulator of gene expression in *Bacillus* (Weng et al., 2013). Other genes, involved in expression of antimicrobial compounds can also be targeted. In *B. subtilis*, the response regulator DegU and its cognate kinase, DegS, constitute a two-component system that regulates many cellular processes, including exoprotease production and genetic competence. Phosphorylated DegU (DegU-P) activates its own promoter and is degraded by the ClpCP protease (Ishii et al., 2013). In plant associated FZB42 the global transcriptional regulator gene *degU* was shown to control non-ribosomal synthesis of secondary metabolites, such as the antifungal lipopeptide bacillomycin D (Koumoutsi et al., 2007), and the antibacterial bacilysin (Mariappan et al., 2012), in FZB42. In an interesting study Xu et al. (2014) demonstrated that stepwise phosphorylation of DegU in *B. amyloliquefaciens* SQR9 can influence BC activity by coordinating multicellular behavior and regulating the synthesis of lipopeptide and polyketide antibiotics in a different manner. Results from *in vitro* and *in situ* experiments and quantitative PCR (qPCR) studies demonstrate that unphosphorylated DegU achieved by a knock out mutation of the *degQ* kinase gene impairs complex colony architecture, biofilm formation, colonization activities, and BC efficiency of *Fusarium* wilt disease but increases the production of the polyketides macrolactin and bacillaene. By contrast, enhanced DegU_P production achieved by *degQ* and *degSU* overexpression does significantly improve complex colony architecture, biofilm formation, colonization activities, production of the antibiotics bacillomycin D and difficidin, and efficiency of BC of *Fusarium* wilt disease.

The transcriptional levels of genes involved in biofilm formation, *yqxM* and *epsD*, were evaluated in response to organic acids via quantitative reverse transcriptase polymerase chain reaction (qRT-PCR). Results suggested that root exudates containing the OAs both induced the chemotaxis and biofilm formation in *B. amyloliquefaciens* NJN-6 (Yuan et al., 2015).

In summary, research with knock-out mutants deepens our knowledge about molecular reasons for the strong antimicrobial activity observed in FZB42 and might contribute to a more efficient use, however, our final goal, developing of biopesticides with constant and enhanced efficiency against plant pathogens needs further, more direct, efforts.

## Pgpr Bacilli Engineered for Enhanced Efficiency in Biocontrol

It is generally assumed that suppression of plant pathogens by PGP Bacilli is based on two features: (1) production of antimicrobial secondary metabolites and siderophores (‘direct antibiosis’), and (2) stimulation of induced systemic resistance (ISR), which activates the plant defense system against harmful microbes and viruses. According to latest results, it is likely that ISR is more important than direct antibiosis in suppressing plant pathogens under conditions of plant rhizosphere. It is very unlikely that concentration of antibiotics within the plant rhizosphere reach levels sufficient for direct antibiosis (Nihorimbere et al., 2012; Chowdhury et al., 2015a,b). Stimulation of ISR is a multifactorial process probably dependent on the presence of several compounds produced by the rhizobacteria, such as the c-LP surfactin and volatiles (Raaijmakers et al., 2010). A strong correlation between the amount of surfactin produced and the ability to elicit ISR was demonstrated (Cawoy et al., 2014). In order to combine both suppressive mechanisms (SR and direct antibiosis), it might be necessary that improved bioformulations contain living *Bacillus* spores and concentrated culture supernatants with antimicrobial metabolites. Besides the number of living spores, also concentration of the main antagonistic metabolite (e.g., bacillomycin D) should be indicated in such formulations (Borriss, 2015b).

It has been proposed early (Compant et al., 2005) to create transgenic PGPB strains that combine multiple mechanisms of action (Timms-Wilson et al., 2000; Chin-A-Woeng et al., 2001; Huang et al., 2004). For example, transforming the 1-aminocyclopropane-1-carboxylic acid deaminase gene, which directly stimulates plant growth by cleaving the immediate precursor of plant ethylene (Glick et al., 1998) into *P. fluorescens* CHAO, not only increases plant growth but can also increase BC properties of PGPB (Wang et al., 2000).

Some studies have demonstrated that the production of cLPs in *Bacillus*, for example, mycosubtilin and iturinA, representatives of the iturin family with antifungal action, and surfactin could be improved by applying promoter exchange strategies.

### Promoter Modulation to Promote Antibiotic Production and ISR

Enhancement of mycosubtilin production in *B. subtilis* strain ATCC 6633 was obtained by replacement of the native promoter of the mycosubtilin operon by a constitutive promoter originating from the replication gene *repU* of the *Staphylococcus aureus* plasmid pUB110. The recombinant strain, designated BBG100, produced up to 15-fold more mycosubtilin than the wild type produced. When tested for its BC potential, wild type strain ATCC 6633 was almost ineffective for reducing a *Pythium* infection of tomato seedlings. However, treatment of seeds with the BBG100 overproducing strain resulted in a marked increase in the germination rate of seeds. This protective effect afforded by mycosubtilin overproduction was also visualized by the significantly greater fresh weight of emerging seedlings treated with BBG100 compared to controls or seedlings inoculated with the wild type strain (Leclère et al., 2005). Enhanced mycosubtilin production (880 mg L^-1^) was also obtained by introducing the tightly regulated *xylA* promoter in front of the *myc* operon of *B. subtilis* ATCC 6633 (Fickers et al., 2009). The P_repU_ promoter was previously reported to enhance the biosynthesis of iturin A, by about threefold in *B. subtilis* RB14 (Tsuge et al., 2001).

The biosurfactant surfactin, a cyclic heptapetide containing four leucine moieties, is known as elicitor of the plant response against pathogens and for its antiviral and antimycoplasmic action (Jacques, 2011). The inducible promoter P_spac_ was used to enhance production of surfactin in *B. subtilis*. After IPTG induction the recombinant *B. subtilis* fmbR-1 produced about 10-fold more than the wild type strain (Sun et al., 2009). In a more sophisticated approach it was found recently, that *comQ* null mutant strains of *B. subtilis* impaired in a social process called quorum sensing (QS), were able to overproduce surfactin. However, overproduction of the secondary metabolite led to reduced fitness of the mutant strains (Oslizlo et al., 2014).

The volatile 2,3-butanediol is known to stimulate ISR in plants (see above). Enhanced production of the volatile in *B. subtilis* was recently demonstrated (de Oliveira and Nicholson, 2015). The genes *alsS*, *alsD*, and *bdhA* encoding acetolactate synthase, acetolactate decarboxylase, and butanediol dehydrogenase, respectively were engineered into a single tricistronic operon under control of the isopropyl β-D-1-thiogalactopyranoside (IPTG)-inducible P_spac_ promoter.

### Modifying Precursor Production

Coutte et al. (2010) hypothesized that precursors supply is one of the main parameters to optimize surfactin production. In fact, overproduction of surfactin was obtained by replacing the native promoter of the surfactin operon *(srfA)* by a constitutive one and disrupting the plipastatin (fengycin) operon *(ppsA)* to save the precursor availability. In order to enhance production of the surfactin precursor leucine, six knockouts were introduced in *B. subtilis* leucine metabolism to verify their effects on surfactin production. For all generated mutants, the specific surfactin production was found increased from 1.6- to 20.9-fold during the exponential growth phase, depending on the medium composition (Coutte et al., 2015). The highest increase in surfactin production was obtained in *codY* mutant strains. This is feasible, since the expression of the *ilv-leu* operon is regulated by CodY in the presence of branched chain amino acids (Ratnayake-Lecamwasam et al., 2001).

## Reconstitution of Product Production

The lanthionine containing bacteriocin mersacidin is not synthesized in FZB42, but an incomplete *mrs* gene cluster presumably directing immunity against the bacteriocin was detected in the genome (**Table 1**). By contrast, the mersacidin producer strain *B. amyloliquefaciens* subsp. *plantarum* HILY harbors the complete *mrs* gene cluster including the genes for synthesis, modification and regulation. In a first step in order to achieve mersacidin production in FZB42, genomic DNA of *Bacillus* HILY mutant strain Rec1 was used to transform competent FZB42 cells. The resulting FZB42 mrs1 strain contained the complete *mrs* gene cluster except the essential genes *mrs*A and *mrs*R1. The completion of the mersacidin gene cluster in FZB42 was achieved *in trans* by transformation with the plasmid pPAR1, carrying the structural gene *mrs*A and *mrs*R1, yielding *B. amyloliquefaciens* mrs1 pPAR1. This surrogate FZB42 derivative was shown to produce active and fully modified mersacidin suggesting that FZB42 can be exploited as an appropriate *in vivo* expression system for the construction and expression of mersacidin analogs (Herzner et al., 2011).

## Modification of Global Regulator

In the following we describe examples for obtaining more powerful strains by applying genetic engineering techniques in the plant-growth-promoting strain FZB42. This work has been performed in the laboratory of Xuewen Gao, Nanjing Agriculture University, China. We have to acknowledge, that at present, use of such engineered PGPR strains under field conditions is refused by the public, at least in Europe. However in light of a steadily increasing world’s population growing from 7 billion now to 8.3 billion in 2025 (Lugtenberg et al., 2013), innovative approaches for getting higher harvest yields without using increasing amounts of agrochemicals should not longer be excluded, given that their use is safe and without harmful consequences for human beings and nature. Careful environmental studies are a precondition before releasing genetic engineered bacteria into the environment (Broer, 2015).

We showed that bacilysin production is strictly controlled by the global regulator DegU (Mariappan et al., 2012). Although bacilysin has potential applications (see above), its use is restricted by low productivity of the producer strains including FZB42. To date, there have been some attempts to increase bacilysin production. However, most experimental approaches were primarily focused on optimizing culture conditions and did not affect basic genetic structures. Ozcengiz et al. (1990) reported that the nitrogen source controls bacilysin biosynthesis, and aspartate was better than glutamate as the sole nitrogen source. Inaoka and Ochi (2011) showed that addition of scandium to the growth medium stimulated the production of bacilysin at the transcriptional level.

**FIGURE 4:**
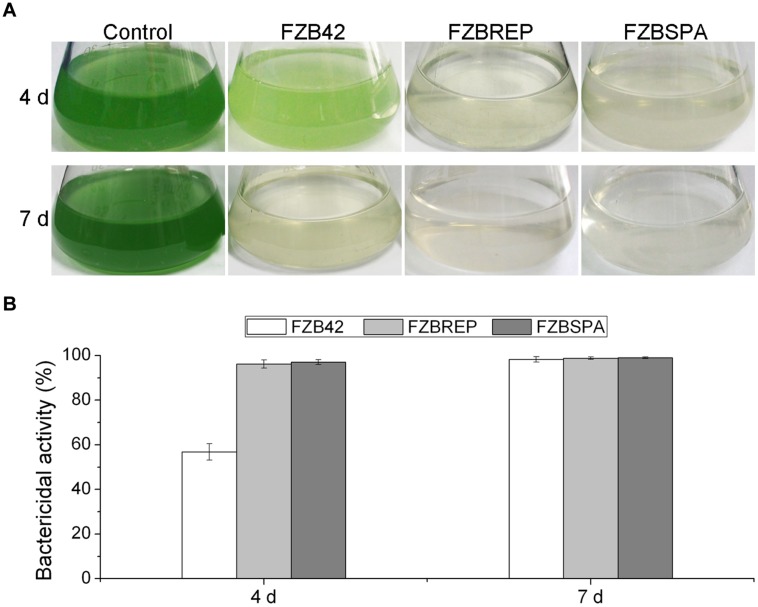
**Suppression of *Microcystis aeruginosa* by transgenic strains FZBREP and FZBSPA.** **(A)** The antagonistic effect of *B. amyloliquefaciens* strains against a liquid *M. aeruginosa* culture after 4 and 7 days. **(B)** The bactericidal activity of *B. amyloliquefaciens* strains FZB42, FZBREP and FZBSPA. Note that suppressive effect of FZBREP and FZBSPA is enhanced compared to wild type FZB42.

In order to improve the production of bacilysin by genetic engineering, Wu et al. (2015b) developed a fast and accurate approach by combining the Cre/*lox* site-specific recombination system with PCR for replacement of the native bacilysin operon promoter with constitutive promoters P*_*repB*_* and P*_spac_* from plasmids pMK3 and pLOSS, respectively. In this system, cre-mediated recombination leads to excision of any DNA (e.g., an antibiotic resistance cassette) in between two distant intramolecular lox sites with a collinear orientation, leaving only one lox site behind and reinstating the antibiotic sensitivity of the respective strain. The engineered markerless strains FZBREP and FZBSPA, expressing the *bac* cassette under the control of the constitutive promoters P*_repB_* and P*_spac_*, significantly increased expression of the *bac* genes, as shown by RT-PCR and qRT-PCR. HPLC confirmed that FZBREP and FZBSPA strains produced up to 170.4 and 315.6 % more bacilysin than wild type, respectively. At 4 days after the *M. aeruginosa* culture had been treated with FZBREP and FZBSPA culture filtrates, the bactericidal activity was >95%, while that of FZB42 was just 56.9% (**Figure 4**). Bacilysin overproduction was also accompanied by enhancement of the antagonistic activities against *S. aureus* (an indicator of bacilysin) and *Clavibacter michiganense* subsp. *sepedonicum* (the causative agent of potato ring rot) (Wu et al., 2015b).

## Foreign Protein Expression in *B. amyloliquefaciens* FZB42

The harpin protein group, which is secreted by many plant pathogenic bacteria during infection, elicits multiple plant responses, resulting in multiple beneficial effects on crop improvement. The *hrp* (“harp”) genes encode type III secretory proteins enabling many phytopathogenic bacteria to elicit a hypersensitive response (HR) on non-host or resistant host plants and induce pathogenesis on susceptible hosts. The HR is a rapid localized death of the host cells that occurs upon pathogen infection and, together with the expression of a complex array of defense-related genes, is a component of plant resistance. The plant genes create a cascade of effects which promote a Systemic Acquired Resistance (SAR) throughout the plant. Beneficial effects on plant growth and health have been reported (Alfano and Collmer, 2004). The protein HrpN_Ea_ produced in *E. amylovora* was the first found and identified in bacteria (Wei et al., 1992). Another hrp gene product, HpaG_Xooc_, from rice pathogenic bacterium *X. oryzae* pv. *oryzicola*, contains two glycine-rich motifs and one cysteine residue (Wu et al., 2009). Despite there are some differences in the sequence and component of amino acids, it exhibits similar biological functions as HrpN_Ea_ protein (Qiao et al., 2014).

The gene *hpa1_Xooc_* encoding protein HpaG_Xooc_ had been cloned onto the expression plasmid pM43HF in *B. subtilis* OKB105, a derivative of *B. subtilis* 168 which is able to produce surfactin and to colonize plant roots (Wu et al., 2009). The engineered strain OKBHF expressing HpaG_Xooc_ protein caused plants to have less severe disease symptoms after inoculation with *Ralstonia solanacearum*, suggesting that HpaG_Xooc_ improves BC efficiency of *B. subtilis* (Gao et al., 2013). However, after 100 generations, the HpaG_XooC_ expression plasmid pM43HF is segregationally unstable in *B. subtilis* under the non-selective condition, thus affecting the continuing expression of HpaG_XooC_ and finally fails to secrete the protein. Transgenic tobacco plants expressing the *hpa1_Xooc_* gene were constructed, but were found unable to induce hypersensitive cell death (HCD) (Peng et al., 2004).

**FIGURE 5:**
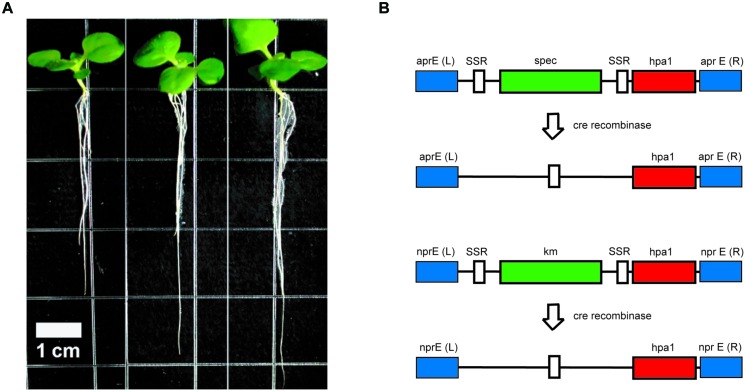
**Strain construction and enhanced root development of tobacco plants by FZBHarpin.** **(A)** Root development of tobacco seedlings is enhanced after treatment with FZB42 and FZBHarpin. From left to right: control, FZB42, FZBHarpin. **(B)** Integration of two copies of the *hpa1* gene (shown in red) into the chromosome of FZB42 via homologous recombination leads to disruption of the genes encoding alkaline (top) and neutral (bottom) protease. The selectable genes, shown in green, are the resistance genes for spectinomycin (spec) and kanamycin (km). White regions represent recognition sites of site-specific recombinases, and blue regions represent sequences homologous between the genome and the integration cassette. Removal of the selectable marker genes is performed with site specific Cre recombinase at the SSR recognition sites.

In order to overcome these difficulties, we decided to use FZB42 as the host strain (Qiao et al., 2013). Two copies of the *hpa1_Xooc_* genes were introduced into the two main extracellular protease genes *apr* and *npr* located on the FZB42 chromosome for avoiding proteolytic destruction of the recombinant harpin gene product (**Figure 5**). RT-PCR analysis showed that the *hpa1_Xooc_* was transcribed. Supernatant of the resulting recombinant strain FZBHarpin caused a hypersensitive response (HR) reaction on tobacco leaf, suggesting biological active Harpin protein is secreted into the medium. The *in vivo* effect of FZBHarpin on plant growth was tested by soaking rice and tobacoo seeds in the suspensions. A significant increase in shoot fresh weight and root length was observed compared to the untreated control and FZB42. Moreover, greenhouse experiments revealed that the control efficacy of FZB42Harpin against rice bacterial blight was 56.9%, showing significant improvement in resistance to *X. oryzae* pv. *oryzicola* relative to FZB42. In addition, a PGP effect by FZB42Harpin exceeding that of FZB42 was also detected (Qiao et al., 2014). However, before applying the recombinant FZB42Harpin strain in field trials, removal of the two resistance markers flanked by the cre *lox* recombinase recognition sites *via* site-directed recombination has to be performed.

## Marker Removal Strategies in *Bacillus*

Classical chromosomal modification is connected with the insertion of a selectable marker, usually a drug resistance gene, into the chromosome of a bacterial strain. Using this strategy, a second selective marker gene is required to introduce another chromosomal modification, so the number of available selection genes limits the feasibility of multiple chromosomal modifications. Moreover, the selectable gene should be removed by single-crossover recombination if the strain is used for further genetic manipulation. In addition, the chance of obtaining a positive strain is relatively low, and the selection process is laborious. To overcome these problems, methods that can eliminate marker cassettes in the primary transformants are needed (Dong and Zhang, 2014). More important, in order to obtain a higher acceptance for genetic engineered strains in agriculture using markerless transgenic strains is a *conditio sine qua non*. Construction of a bacilysin overproducing FZB42 strain described above is an example for successful application of this technique in plant-associated *Bacillus* strains.

Today there are several methods for marker removal available. Site-specific recombination (SSR) systems are capable of eliminating antibiotic resistance markers, if they are flanked by recombinase recognition sites. SSR systems that are used in *B. subtilis* are Cre/loxP from bacteriophage P1, and Xis/attP from bacteriophage λ.

In a previous study (Leibig et al., 2008), a Cre-*lox* setting was established that allowed the efficient removal of resistance genes from the genomes of *S. carnosus* and *S. aureus*. Two cassettes conferring resistance to erythromycin or kanamycin were flanked with wild type or mutant *lox* sites, respectively, and used to delete single genes and an entire operon. After transformation of the cells with a newly constructed *cre* expression plasmid, genomic eviction of the resistance genes was observed in approximately one out of ten candidates analyzed and subsequently verified by PCR. Due to its thermo-sensitive origin of replication, the plasmid can be eliminated at non-permissive temperatures and marker-less deletion mutants can be obtained.

Although Cre-mediated recombination and excision of the chromosomal sequence between two lox sites is efficient, it does not occur in all cells. To address this, Wang et al. (2012) developed a simple and efficient *B. subtilis* genome editing method in which targeted gene(s) could be inactivated by single-stranded PCR product(s) flanked by short homology regions and in-frame deletion could be achieved by incubating the transformants at 42°C. In this process, homologous recombination was promoted by the lambda beta protein synthesized under the control of promoter PRM in the lambda cI857 PRM–PR promoter system on a temperature sensitive plasmid pWY121. The hen egg white lysozyme gene is placed after promoter PR, which is effective in *B. subtilis*, and is precisely regulated by the CI857 repressor protein (Breitling et al., 1990). The efficiency of inframe deletion using this method can reach 100%. As hen egg white lysozyme is active against *Bacillus* species, and its encoding gene is distantly related to *Bacillus* genes, it could also be effective in other *Bacillus* species.

## Conclusion

Due to increasing environmental problems caused by the exaggerated use of chemicals in agriculture, further improvement of BC agents is a timely task. Possibilities for developing more efficient bioagents include comparative genomic analysis in order to detect specific features unique for plant-associated bacteria and their improvement by applying genetic methods. Due to their ability to produce durable endospores plant-beneficial *Bacillus* strains offer perfect possibilities for stable bioformulations, which are competitive to the widely used agrochemicals. In order to enhance progress in getting highly efficient bioformulations, we have proposed to focus further research about plant-bacteria interactions on the model bacterium *B. amyloliquefaciens* FZB42, which is genetic amenable, widely used in practice, and in which a huge knowledge base already exists.

In this review we present examples for engineering several features, important for suppression of plant pathogens by direct antibiosis and ISR. Strategies applied include (1) modulation of promoter activity, (2) modification of precursor production, (3) reconstitution of product production, (4) control of metabolite production by global regulators, and (5) expression of foreign proteins. Although examples for applying such genetic engineering strategies in spore-forming Bacilli are relatively scarce, it is to expect that they will become in future a powerful tool for further improvement of biopesticides and biofertilizer, given that the public will change their behavior against use of engineered environmental bacteria.

## Outlook

Today, applying and release of genetic engineered bacteria directly in the environment is not accepted by the public and governmental regulations are contradictory for use of such microorganisms in enhancing crop yield. One reason is the presence of resistance genes in transgenic strains, which have been introduced in the bacteria during the allelic replacement process, and methods avoiding use of such marker genes are therefore highly desirable.

Of course, marker removal is not the only precondition for improved acceptance of genetically engineered strains when released into the environment. As stated above, careful case studies demonstrating that no harmful effects caused by genetic engineered strains are urgently needed. In applying genetic engineered plant growth promoting bacteria we have to distinguish two different levels:

(1) Engineered strains without foreign genes but containing useful mutations in genes affecting the beneficial effect of the bacterium in terms of plant growth-promotion and BC of pathogens. Given that no resistance marker has been introduced, it might be unimportant whether the useful mutation has been introduced by a targeted allele exchange or has been evolved after applying a natural selection procedure. We believe, that such strains will be accepted in future when their improved action has been convincingly demonstrated.
(2) Engineered strains containing genes from other bacteria. Such bacteria will be considered as “recombinant,” also when the donor bacteria occur in the same natural environment. This was the case in the example described here. Ironically, the harpin gene isolated from a pathogen bacterium was shown to act beneficial when cloned and expressed in FZB42. However, long-term environmental studies are necessary to demonstrate that such recombinant bacteria do not harm environment by novel recombination events with other microorganisms occurring in the same environment.

In summary, genomic analysis is already an important tool in characterizing of beneficial microbes. Moreover, we believe on the prospect of genetic engineering for obtaining improved bioformulations in future. This development should contribute to a more sustainable agriculture, and enabling us to save considerable amounts of agrochemicals, such as chemical fertilizers and chemical pesticides.

## Conflict of Interest Statement

The authors declare that the research was conducted in the absence of any commercial or financial relationships that could be construed as a potential conflict of interest.

## Footnotes

1 www.bccresearch.com/market-research/chemicals/biopesticides-chm029e.html

2 http://www.nordreet.de/bacillus-consulting/literatur/

3 http://www.abitep.de/de/produkte.html

4 http://www.nordreet.de/bacillus-consulting/research/
